# An Infrequent Complication of VT Ablation: Should We Perform Delayed Routine Imaging After Steam Pops?

**DOI:** 10.1016/j.jaccas.2022.01.006

**Published:** 2022-06-01

**Authors:** Ez Alddin Rajjoub Al-Mahdi, Sara Fernández Santos, José López-Menéndez, Covadonga Fernández-Golfín, Javier Moreno

**Affiliations:** a Cardiology Department, University Hospital Ramon y Cajal, Madrid, Spain; b Cardiovascular Surgery Department, University Hospital Ramon y Cajal, Madrid, Spain

**Keywords:** ablation, complication, ventricular tachycardia, CMR, cardiac magnetic resonance, RF, radiofrequency

## Abstract

Catheter ablation has become the cornerstone of the treatment of ventricular arrythmias. Nevertheless, it is crucial to recognize the adverse effects of such treatment. We present a case of an incidental diagnosis of ventricular pseudoaneurysms after catheter ablation for treatment of drug-refractory nonsustained runs of ventricular tachycardia. (**Level of Difficulty: Advanced.**)

## Central Illustration

**Figure:**
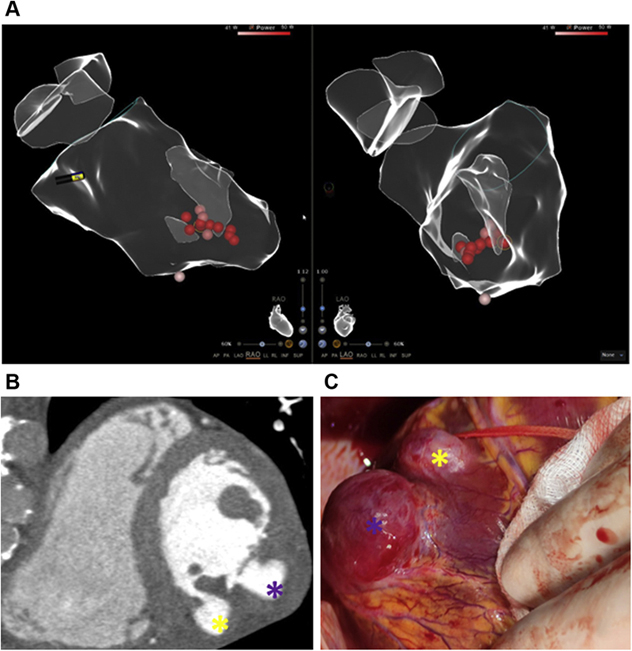

A 51-year-old-man was admitted to our center because of drug-refractory incessant repetitive nonsustained ventricular tachycardia. The patient described having palpitations, but no other symptoms were present. At arrival, blood pressure was 100/60 mmHg, pulse was irregular, oxygen saturation on room air was 99%, and respiratory rate was 18. Physical examination showed isolated pectus excavatum. Nevertheless, he did not meet the criteria for the diagnosis of any connective tissue disease associated with pectus excavatum. Cardiac auscultation did not reveal murmurs, and no signs of heart failure were found.

A baseline electrocardiogram showed sinus rhythm, normal PR interval, narrow QRS, and normal repolarization with frequent monomorphic ventricular ectopics and nonsustained ventricular tachycardia (with right axis deviation, right bundle branch block—like morphology, and negative transition in V_5_) (Supplemental Figures 1 and 2). An echocardiogram showed a normal left ventricle, but significant pectus excavatum limited proper characterization of the right ventricle. Valvular morphology and function and aortic root were normal. Cardiac magnetic resonance (CMR) did not reveal significant findings, but the study quality was limited because of the ventricular arrhythmia. Thus, it was recommended to repeat CMR after ablation.

**Figure 1:**
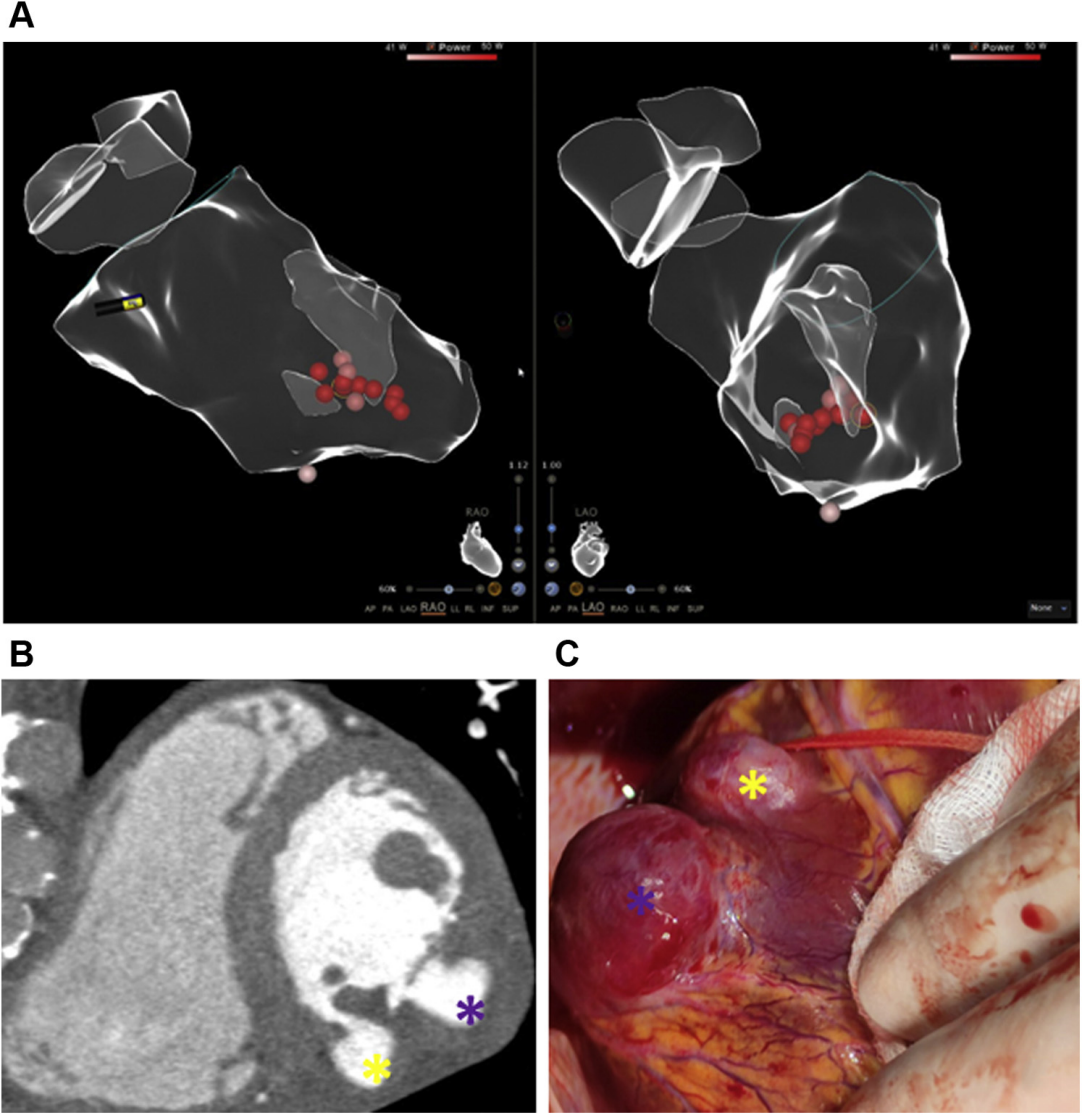
Ablation Dots and Pseudoaneurysms Location **(A)** Left ventricle anatomical map with right anterior oblique and left anterior oblique projections, showing the 2 areas where radiofrequency was applied. **(B)** Short-axis CT scan showing inferior pseudoaneurysm **(yellow asterisk)** and inferolateral pseudoaneurysm **(purple asterisk)**. **(C)** Surgical view.

Catheter ablation was guided by CartoSound electroanatomical mapping and intracardiac echocardiography, using a combined retroaortic and transeptal approach (Figure 1A). Activation mapping of clinical ectopies was performed with a PentaRay catheter, and the site with the earliest potentials (-18 ms) was located at the middle third of the inferior LV aspect, between the bases of both papillary muscles. Local radiofrequency (RF) applications successfully terminated the clinical ventricular premature beats (ThermoCool SmartTouch, normal saline irrigation, 40 to 50 W, 50 to 60 seconds, average contact force 12 g). The last bonus application was interrupted prematurely after 37 seconds because of a steam pop. Supplemental Figure 3 shows trends during this application, with marked impedance drop (>20 ohm) and 2 impedance sharp rises before steam pop. Note that only the second rise was associated with temperature increase, which was probably the cause of the steam pop.

A new reproducible morphology of ventricular ectopics appeared (left superior axis, right bundle branch block type), and they were targeted. Their origin was located at the middle third of the inferoseptal region of the LV, and a single RF application terminated them (41 W, 88 seconds, average contact 9 g). Supplemental Figure 4 shows no dangerous impedance trend during this application. The patient was discharged 2 days later without ventricular tachycardia or ectopies.

Three months later, the patient was asymptomatic, and a Holter recording did not reveal remarkable arrhythmias. CMR was repeated in the attempt to definitively exclude structural heart disease. Unexpectedly, it showed 2 pseudoaneurysms at both areas where RF had been applied (Supplemental Figure 5, Video 1). Computed tomography ruled out coronary artery disease and confirmed the CMR findings: an inferior pseudoaneurysm with a maximum diameter of 15 mm and a neck of 7 mm, and an inferolateral pseudoaneurysm with a maximum diameter of 20 mm and a neck of 9 mm (Figure 1B). The patient underwent semiurgent surgical repair (Figure 1C). The pseudoaneurysms were directly opened and subsequently closed by a 2-layer approach: the inner aspect was closed by a Prolene 5/0 running suture, and the external aspect by a double layer of Teflon strips and Prolene 4/0 suture. Five days later, the patient was discharged home uneventfully.

This case highlights the possible occurrence of ventricular pseudoaneurysm as an extremely rare complication after RF ablation, with only very scarce previous reports available.^1^ Interestingly, to our knowledge this is the first reported case of 2 pseudoaneurysms after a catheter ablation procedure. Owing to the clinical scenario, high-power parameters were set up, because catheter stability may be challenging to maintain in papillary tachycardia. If lower power had been applied, successful lesions might have been achieved likewise, but reducing the risk of steam pop. It has been previously reported that 80% of pops occurring during ventricular tachycardia ablation are preceded by an impedance decrease of ≥18 ohm.^2^ A ventricular pseudoaneurysm may be asymptomatic; it portends a clinical problem whether it should be routinely excluded after catheter ablation, given that at least 1 case of lethal rupture has been reported.^1^ As a life-threatening complication, routine imaging 2 to 3 months after ablation may be indicated after ventricular ablation, particularly if steam pop is detected.^3^

## Appendix

Supplemental Video 1 Cardiac magnetic resonance: cine sequence showing left ventricular pseudoaneurysms.
