# Supplementary material for: An Infrequent Complication of VT Ablation: Should We Perform Delayed Routine Imaging After Steam Pops?
Source: JACC Case Rep. 2022 Jun 1;4(11):655–7. doi: 10.1016/j.jaccas.2022.01.006 (PMC9168779; doi:10.1016/j.jaccas.2022.01.006)
Supplement: Supplemental Figures 1–5 [file mmc2.docx]

**
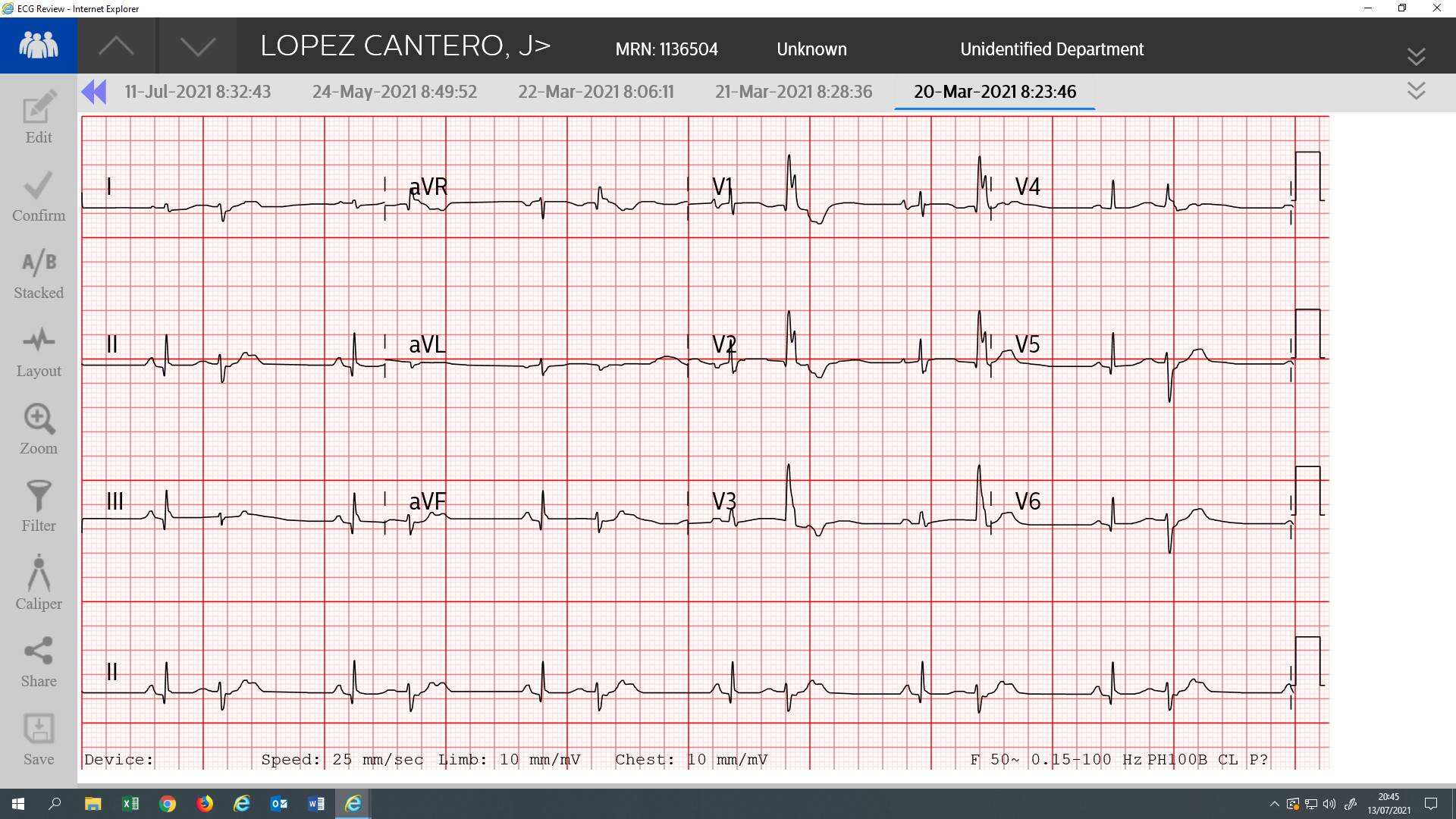
SUPPLEMENTAL FIGURE 1. Electrocardiogram in sinus rhythm and ventricular bigeminy. Ectopics show right axis deviation, RBBB-like morphology and negative transition in V5.**

**
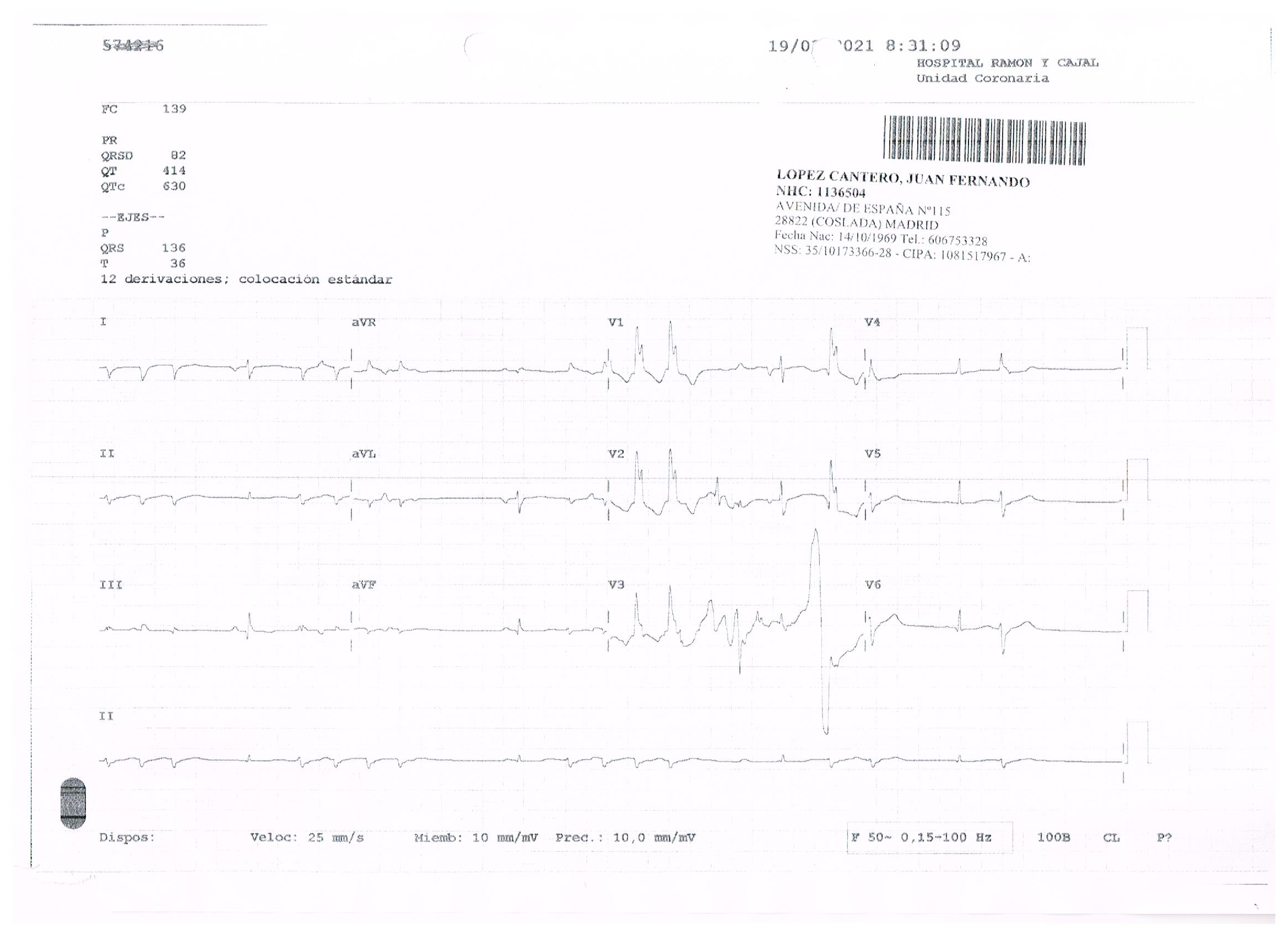
SUPPLEMENTAL FIGURE 2. Incessant repetitive NSVT.**

**SUPPLEMENTAL FIGURE 3. Impedance, contact force and power trends during last bonus application in PVC 1 region (pop). Note the marked impedance drop (> 20 ohm). In addition, it can be appreciated steep impedance rises.**


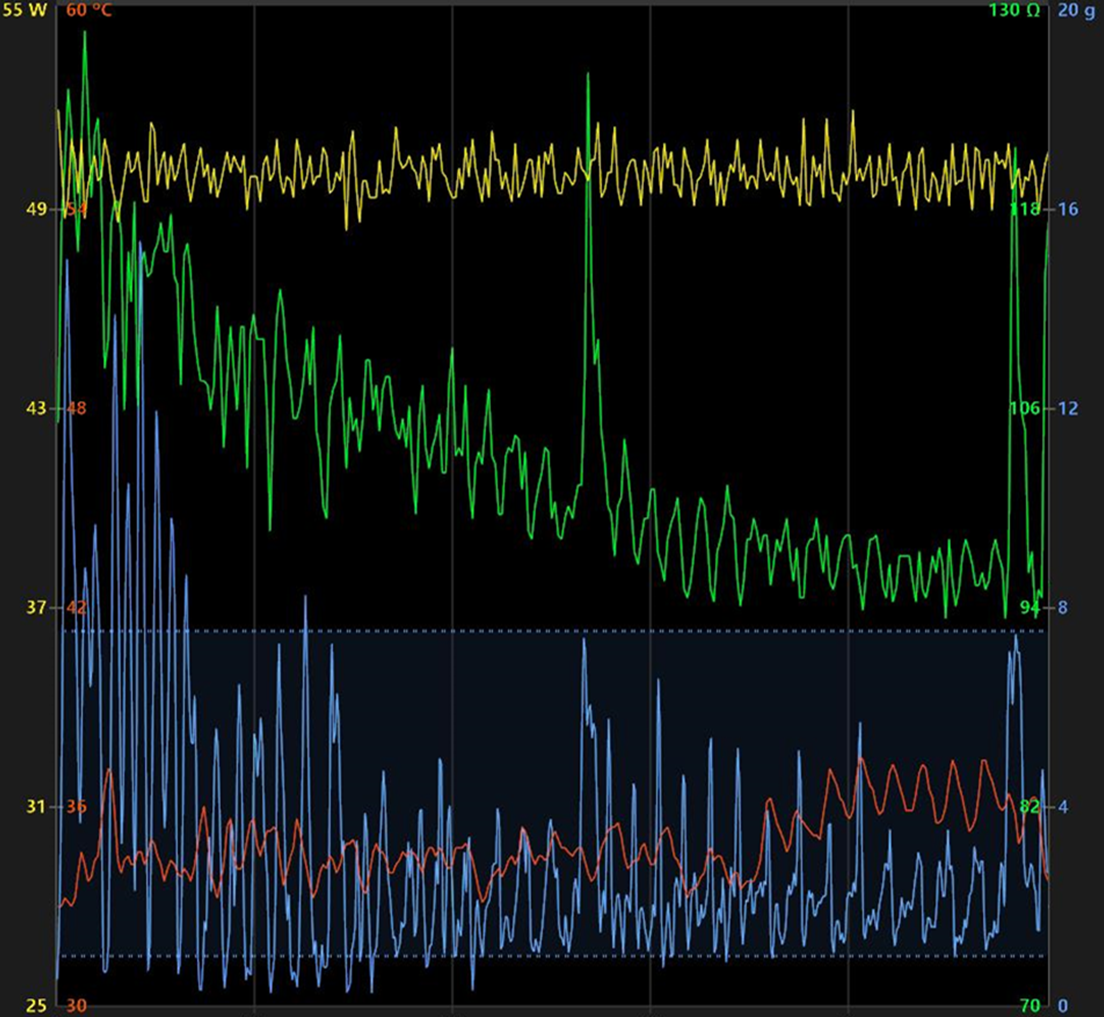


**SUPPLEMENTAL FIGURE 4. Impedance, contact force and power trends during PVC 2 application. No dangerous impedance or temperature were noted.**


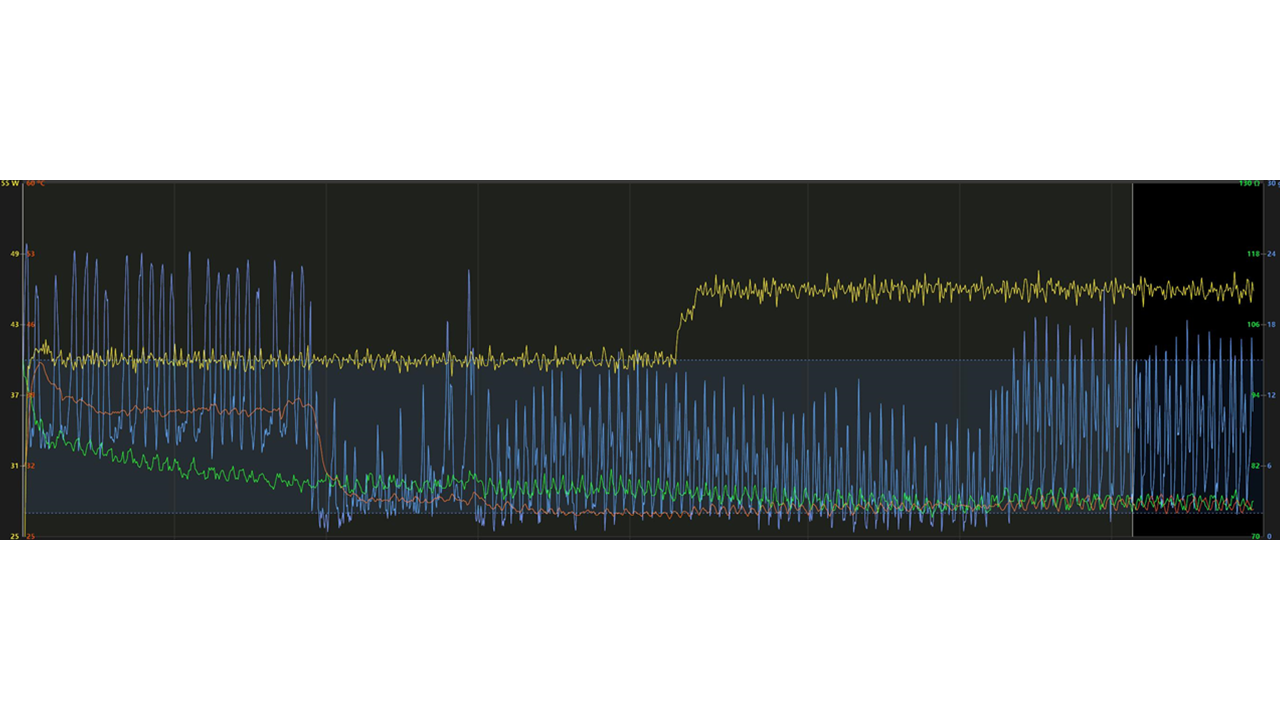


**
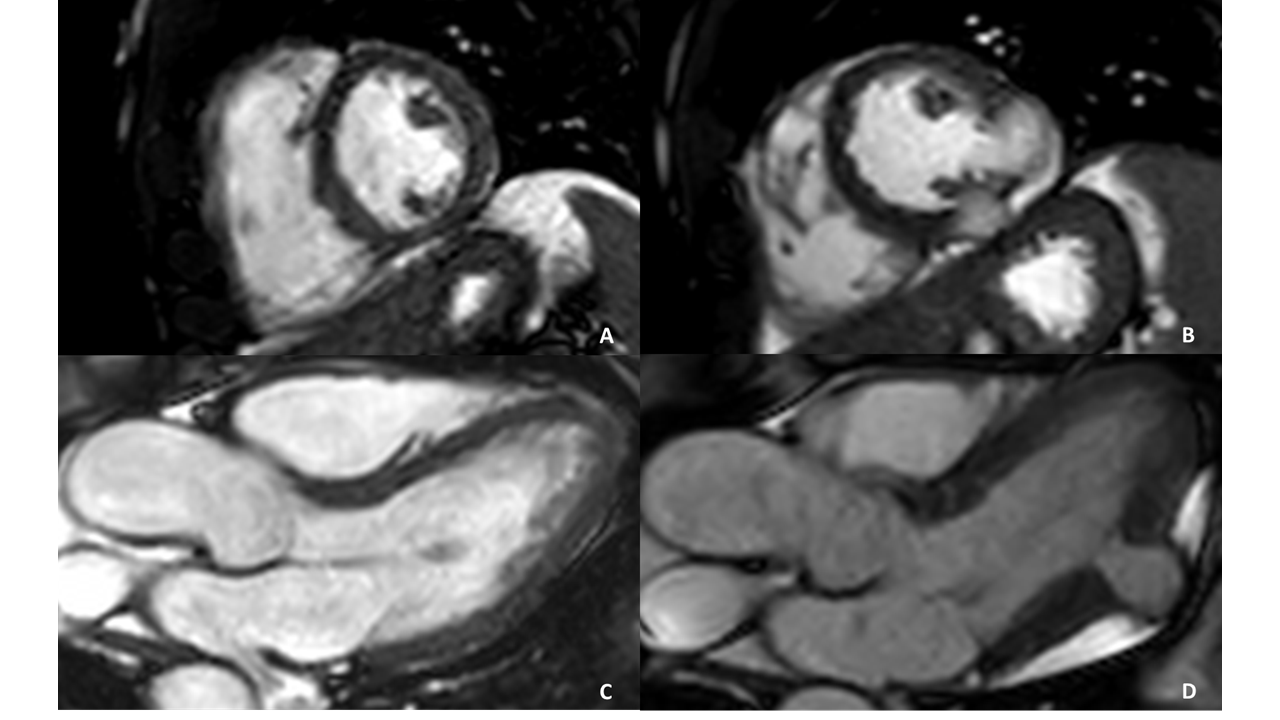
SUPPLEMENTAL FIGURE 5. Cardiac magnetic resonance. A and C panels: pre-ablation short and long LV axis (three-chamber view), respectively; B and D panels, similar post-ablation short and long LV axis (three-chamber view), showing pseudoaneurysms.**
